# Subjective Well‐Being and Its Predictors in Parkinson's Disease and Dystonia: A Comparative Study

**DOI:** 10.1002/mdc3.70141

**Published:** 2025-05-30

**Authors:** Suzette Shahmoon, Dejan Georgiev, Paul Jarman, Kailash Bhatia, Patricia Limousin, Marjan Jahanshahi

**Affiliations:** ^1^ Department of Clinical and Movement Neurosciences UCL Queen Square Institute of Neurology London UK; ^2^ Department of Neurology University Medical Centre Ljubljana Ljubljana Slovenia; ^3^ Faculty of Computer and Information Sciences University of Ljubljana Ljubljana Slovenia

**Keywords:** dystonia, Parkinson's disease, positive psychology, self‐esteem, subjective well‐being

## Abstract

**Background:**

Quality of life (QoL) is a commonly used outcome measure in people with chronic neurological diseases (CND). As valuable as QoL is, it does not take into account aspects of subjective well‐being (SWB) such as subjective happiness, meaning in life, life satisfaction and hope; all constructs that are considered central to well‐being.

**Objectives:**

The goal was to assess how the different aspects of SWB are altered in Parkinson's disease (PD) and dystonia relative to healthy controls (HCs) and to identify the most important predictors of different dimensions of SWB in PD and dystonia.

**Methods:**

Eighty‐two people with PD, 63 with dystonia, and 50 HCs were surveyed using various measures of SWB.

**Results:**

People with PD and dystonia had significantly lower satisfaction with life than HCs, with self‐esteem and loneliness being significant covariates. Although people with PD and dystonia had significantly less meaning in life, they still sought meaning in life as much as HCs. Self‐esteem, resilience and neuroticism were significant covariates for the presence of “meaning in life.” There were no significant group differences in subjective happiness or hope. In people with PD, mood (depression and anxiety), personality traits (extraversion and self‐esteem), disease duration, and pain/discomfort were significant predictors of various measures of SWB. The main significant predictor of SWB in dystonia was depression, which predicted 49% of the variance in subjective happiness.

**Conclusions:**

These results emphasize the importance of SWB as a field of investigation and clinical care in the management of people with PD and dystonia.

The impact of living with a chronic neurological disease (CND) such as Parkinson's disease (PD) or focal/segmental craniocervical dystonia (FSCD) goes beyond physical well‐being. Quality of life (QoL) assesses how the individuals perceive their lives and is influenced by context, including socioeconomic background and culture.^1^ Health‐related QoL (HRQoL) considers the impact of illness on subjective measures of emotional, physical, and social functioning.^2^ HRQoL in people with PD decreases significantly with the stage and severity of the disease.^3^ Dyskinesias,^4^, ^5^ non‐motor symptoms such as depression,^6^ cognitive dysfunction,^7^ and stigma^8^ also have an impact on HRQoL. Similarly, people with FSCD have a poorer HRQoL than healthy controls (HCs).^9^, ^10^, ^11^ In cervical dystonia, lower HRQoL has been associated with motor symptoms, psychiatric, and behavioral symptoms.^12^

There are now calls to assess subjective well‐being (SWB), which relates more to the embodied emotional state of the individual than to the social construct in which they live.^13^ Although there is no one gold standard for assessment of SWB, most agree that sense of purpose,^14^ happiness, pleasure, low levels of negative mood, and high levels of life satisfaction^15^ predict SWB. In addition, according to “positive psychology,” pleasure, meaning, and engagement with life are the main components of SWB.^16^

There are very few measures of SWB that relate specifically to PD or dystonia. Measures such as the Parkinson's Disease Questionnaire‐39^17^ have assessed HRQoL in PD, but are not designed to evaluate the various aspects of SWB. The closest measure is the challenges and potentials (CHAPO) model as it takes a more holistic approach to understanding the challenges and potentials of living with PD, assessing environmental and individual factors that are objective and subjective.^18^ However, neither of these tools assess components of SWB such as engagement, meaning, or pleasure in life. Furthermore, disease‐specific instruments are useful to illustrate the effects of disease and treatments in clinical trials and in daily life,^19^ but they do not allow for comparisons with HCs. As many instruments have been developed and validated to assess SWB in the general population, including the Subjective Happiness Scale (SHS),^20^ the State Hope Scale (SHoS),^21^ the Meaning in Life Questionnaire (MLQ),^22^ and the Satisfaction with Life Scale (SLS),^23^ the main aim of the study was to investigate the differences in SWB of people with PD and FSCD compared to HCs by the use of these measures. As with HRQoL, we expected PD and FSCD to have a negative impact on SWB, because of the physical, emotional, and role‐related limitations they impose on the individual. Furthermore, different conditions might influence SWB in PD and FSCD. For example, the experience of loneliness has been shown to worsen PD.^24^ Self‐esteem, perceived stigma, depression, anxiety,^25^, ^26^ and degree of optimism^27^, ^28^ can all influence SWB in different ways as can personality traits, which can influence coping strategies when dealing with a chronic disease such as PD and FSCD.^29^ Therefore, we were also interested in identifying the most important predictors of the different dimensions of SWB in PD and FSCD.

## Methods

### Participants

One hundred fifty people with PD were initially contacted, 98 (65%) of whom responded. The final number of people with PD enrolled in the study was 82 (55%). Similarly, 98 people with FSCD were initially contacted, 66 (67%) of whom responded and 63 (64%) were enrolled in the study. All patients were recruited through the outpatient clinics of three collaborating consultant neurologists (P.L., K.B., P.J.) at the National Hospital of Neurology and Neurosurgery, Queen Square, London. The participants had a diagnosis of either idiopathic PD according to the criteria of the United Kingdom (UK) Brain Bank^30^ or FSCD (blepharospasm, cervical dystonia, Meige syndrome).^31^ In addition, 77 HCs were, through social and community networks, initially contacted to participate in the study, 50 (65%) of whom responded and all of them were enrolled in the study. HCs were invited to participate if they had no chronic neurological, psychiatric, or physical diseases and were over 40 years of age. Eligible participants emailed the research team and a questionnaire pack was sent to them.

The sample size required to perform the study was estimated based on a significance level of 0.05, a power of 0.80, and a medium‐high effect size, based on previous research.^32^ This study was anticipated to have a medium effect size of 0.35. Using power statistics, the minimum sample size was estimated to be 57 per group. The minimum number of participants was recruited in each group, except for HCs as recruitment had to stop because of the onset of the coronavirus disease 2019 pandemic.

A booklet containing the scales and questionnaires listed below together with an information sheet explaining the aims of the study was mailed to each participant. As suggested by the Ethics Committee who reviewed and approved the proposal, the return of the completed booklet acted as implied consent. Full ethical approval was granted by Health Research Authority and Health and Care Research Wales Ethics Committee. Full ethics approval for the project was obtained on February 2, 2018, application number 233401, REC Ref 18/LO/1368.

In addition to the demographic and clinical information on age, gender, marital status, type of disease, and disease duration, the following scales and questionnaires (a detailed description of the measures is given in the Data S1). Description of the scales and questionnaires used in the study, were used to assess the subjective and mental well‐being of participants. Measures of subjective well‐being: (1) SHS,^20^ which measures subjective happiness (SH); (2) SHoS^21^ with its two subscales trait hope pathways and trait hope agency; (3) MLQ^22^ with its two subscales meaning in life search score and meaning in life presence score; and (4) SLS,^23^ which measures satisfaction with life (SWL). Generic quality of life measure: EQ‐5D.^33^ Mental health measures: (1) Hospital Anxiety and Depression scale (HADS)^34^ with its two subscale measuring depression and anxiety respectively; and (2) the Univesity of California Los Angeles (UCLA) Loneliness Scale (LS).^35^ Other measures: (1) Self‐Esteem Questionnaire (SEQ);^36^ (2) the Stigma Scale (SS);^37^ (3) Eysenck Personality Questionnaire (EPQ) short form,^38^ which measures neuroticism, extraversion, psychoticism, and lie; (4) Life Orientation Test (LOT);^39^ (5) the Short Social Support Questionnaire (SSSQ);^40^ and (6) the Brief Resilience Scale (BRS).^41^

### Statistical Analysis

IBM SPSS v29 for Mac was used for analysis. The significance level was set at 0.05. A χ^2^‐test was performed to determine whether the proportion of genders was equal between groups.

To assess the differences between PD, dystonia, and HCs in the measures of SWB a series of one‐way analysis of covariance (ANCOVAs) were completed, with group as the between groups factor and a series of potential covariates to control for possible group differences. The covariates included age, gender, anxiety (HADS), depression (HADS), life orientation (LOT), mobility and pain/discomfort items from the EQ‐5D, loneliness (LS), self‐esteem (SEQ), extraversion, and neuroticism from EPQ and resilience (BRS). Independent groups, 2‐tailed *t* tests were used for planned post hoc analysis to further assess the differences between PD, dystonia, and HCs.

To assess which variables (age, gender, disease duration, anxiety [HADS], depression [HADS], mobility and pain/discomfort items from the EQ‐5D, loneliness [LS], self‐esteem [SEQ], life orientation [LOT], extraversion and neuroticism from EPQ, social support stigma [SSSQ], resilience [BRS]) best predicted subjective well‐being in PD and dystonia, a series of multiple regression analyses were carried separately for PD and dystonia patients for each of the measures of SWB. Although the rate of missing values in the variables used in the regression analysis was low, the missing values were replaced by the mean values of the variables before the regression analysis was executed.

## Results

There was no significant difference in age and gender between groups. Participants with FSCD had a longer disease duration compared to PD *P* = 0.001 (Table 1).

**TABLE 1:** Demographic and essential clinical details of the participants in the study

	Parkinson's disease	FSCD	Healthy controls	*P*‐value
Age‐y	68.16 ± 8.34	67 ± 10.44	58.76 ± 12.05	0.586
Gender (M:F)	40:42	23:40	22:28	0.280
Disease duration‐mo	86.04 ± 56.33	293.84 ± 159.80	/	0.001
HADS				
Depression	5.96 ± 3.41	5.88 ± 3.91	3.25 ± 3.54	0.545
Anxiety	7.44 ± 4.33	7.86 ± 4.89	5.78 ± 6.37	0.681
UCLA Loneliness Scale	38.03 ± 10.67	40.96 ± 13.70	38.00 ± 8.75	0.138
Marital status				
Married	59	48	30	
Single	7	8	3	
Separated	1	0	1	
Divorced	8	1	11	
Widow	7	3	1	
Partnership	0	3	4	

*Note*: Age (in y), disease duration (in mo) and the mental health measures (HADS and UCLA Loneliness Scale) are presented as mean ± standard deviation of the mean. Gender and marital status are presented as number of participants per category.

Abbreviations: FSCD, focal/segmental craniocervical dystonia; M, male; F, female; HADS, Hamilton Anxiety and Depression Scale.

### Differences in Measures of Subjective Well‐being between Groups

#### SH

**FIG. 1:**
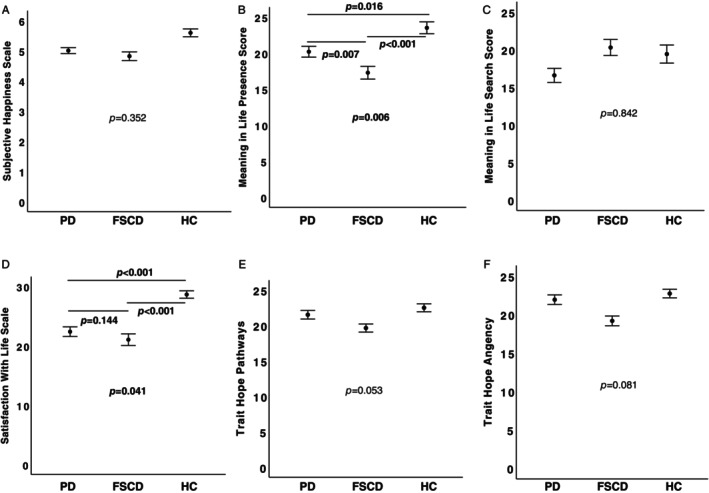
Differences in measures of subjective well‐being in patients with Parkinson's disease (PD), focal/segmental craniocervical dystonia (FSCD), and healthy controls (HC); mean values of Subjective Happiness Scale (**A**), Meaning in Life Presence Score (**B**), Meaning in life Search Score (**C**), Satisfaction With Life Scale (**D**), Trait hope Pathways (**E**) and Trait Hope Agency (**F**) are presented. Error bars represent standard error of the mean. The *P* < 0.05 considered significant are written in bold.

After controlling for the significant effects of resilience (*P* = 0.015), self‐esteem (*P* = 0.035), depression (*P* = 0.007) on SH, there were no significant differences between groups on SH (Fig. 1A).

#### Meaning in Life

After controlling for the significant effect of self‐esteem (*P* < 0.001), resilience (*P* = 0.025), and neuroticism (*P* = 0.049) the effect of group on the presence score was significant (*P* = 0.006) (Fig. 1B). The presence score was significantly lower in both PD (*P* = 0.016) and FSCD (*P* < 0.001) compared to HCs, and it was significantly higher in PD compared to FSCD patients (*P* = 0.007). None of the covariates significantly explained the search score (all *P* > 0.250), which was not significantly different between the groups (Fig. 1C).

#### SWL

After controlling for self‐esteem (*P* = 0.027) and loneliness (*P* = 0.015), the difference between groups in SWL was significant (*P* = 0.041) (Fig. 1D). SWL was lower in both PD (*P* < 0.001) and FSCD (*P* < 0.001) compared to HCs. The difference in SWL between PD and FSCD was not significant.

#### Hope

After controlling for age (*P* = 0.024), self‐esteem (*P* < 0.001), and anxiety (*P* = 0.008), the differences between groups in hope pathways was not significant (Fig. 1E). Similarly, after controlling for the mobility item of EQ‐5D (*P* = 0.016), life orientation (*P* = 0.002), and extraversion (*P* = 0.049), the difference in hope agency between groups was also not significant (Fig. 1F).

### Predictors of Subjective Well‐Being in PD

The results of predictors of subjective well‐being in PD are summarized in Table 2.

**TABLE 2:** Significant predictors of subjective well‐being in Parkinson's disease and focal/segmental craniocervical dystonia

Parkinson's disease			
Subjective happiness scale	*F*(18, 64) = 4.41, *P* < 0.001, adjusted *R* ^2^ = 0.43		
Resilience	β = 0.35	95% CI [0.17–0.74]	*P* = 0.002
Extroversion	β = 0.22	95% CI [0.012–0.11]	*P* = 0.014
Depression	β = −0.44	95% CI [−0.21 to −0.03]	*P* = 0.011
Meaning in life questionnaire search score	*F*(18, 64) = 2.63, *P* = 0.002, adjusted *R* ^2^ = 0.26		
Anxiety	β = 0.42	95% CI [0.07–1.61]	*P* = 0.033
Satisfaction with life scale	*F*(18, 64) = 3.39, *P* < 0.001, adjusted *R* ^2^ = 0.35		
Self‐esteem	β = 0.30	95% CI [0.06–0.96]	*P* = 0.028
Anxiety	β = −0.35	95% CI [−1.10 to −0.09]	*P* = 0.021
State hope scale trait hope pathway	*F*(18, 64) = 4.71, *P* < 0.001, adjusted *R* ^2^ = 0.45		
Disease duration	β = −0.27	95% CI [−0.045 to −0.08]	*P* = 0.005
Self‐esteem	β = 0.52	95% CI [0.34–0.96]	*P* < 0.001
Pain/discomfort	β = 0.33	95% CI [0.55–3.36]	*P* = 0.007
State hope scale trait hope agency	*F*(18, 64) = 4.71, *P* < 0.001, adjusted *R* ^2^ = 0.51		
Disease duration	β = −2.74	95% CI [−0.045 to −0.008]	*P* = 0.005
Self‐esteem	β = 0.52	95% CI [0.34–0.96]	*P* < 0.001
Anxiety	β = −0.36	95% CI [−0.95 to −0.06]	*P* = 0.026

Focal/segmental craniocervical dystonia			
Subjective happiness scale	*F*(17, 45) = 4.45, *P* < 0.001, adjusted *R* ^2^ = 0.49		
Depression	β = − 0.38	95% CI [−0.215 to −0.02]	*P* = 0.019
Satisfaction with life scale	*F*(17, 45) = 2.98, *P* = 0.002, adjusted *R* ^2^ = 0.35		
State hope scale trait hope pathway	*F*(17, 45) = 1.91, *P* = 0.042, adjusted *R* ^2^ = 0.20		
Resilience	β = 0.33	95% CI [0.08–3.11]	*P* = 0.039

#### SH

The regression model predicting SH was significant (*P* < 0.001). Resilience significantly predicted SH (*P* = 0.002), as did extraversion (*P* = 0.014) and depression (*P* = 0.011).

#### Meaning in Life

The regression model predicting the “presence” score was not significant. Nevertheless, the regression model predicting the “search” score was significant (*P* = 0.002). Anxiety significantly predicted the “search” score (*P* = 0.033).

#### SWL

The regression model predicting SWL was significant (*P* < 0.001), with self‐esteem (*P* = 0.028) and anxiety (*P* = 0.021) significantly predicting SWL.

#### Hope

The regression model predicting the hope pathways score was significant (*P* < 0.001) with disease duration (*P* = 0.005), self‐esteem (*P* < 0.001), and pain/discomfort (*P* = 0.007) significantly predicted hope pathways. The regression model predicting hope agency was also significant (*P* < 0.001), with disease duration (*P* = 0.05), self‐esteem (*P* < 0.001) and anxiety (*P* = 0.026) predicted hope agency.

### Predictors of Subjective Well‐Being in FSCD

The results for predictors of subjective well‐being in FSCD are summarized in Table 2.

#### SH

The regression model predicting SH in FSCD was significant (*P* < 0.001), with only depression significantly predicting SH (*P* = 0.019).

#### Meaning in Life

The regression models predicting meaning in life presence and search scores in FSCD were not significant.

#### SWL

The regression model predicting SWL in FSCD was significant (*P* = 0.002), but none of the predictors significantly predicted SWL.

#### Hope

The regression model predicting hope pathway in FSCD was significant (*P* = 0.042), with resilience significantly predicting hope pathway (*P* = 0.039). The model predicting hope agency in FSCD was not significant.

## Discussion

The main findings of the study suggest that there are differences in some, but not all measures of SWB between PD, FSCD, and HC. More specifically, PD and FSCD showed lower SWL and lower meaning in life presence score than HC. The meaning in life presence score was significantly higher in PD compared to FSCD patients. There were no differences between the groups in terms of SH, search for meaning in life, or hope. Although we identified several significant predictors of specific measures of SWB in PD, depression was the only significant predictor of SH and resilience was the only significant predictor of pathways to hope in FSCD.

The set‐point theory suggests^42^ that people have their own individual happiness “set point.” A negative life event or challenge, such as a disease onset, can lower happiness, yet over time, one can overcome the challenge or habituate to difficult circumstances, allowing for people to return to their natural set point of happiness.^42^ This was supported by our findings that there were no differences between groups in terms of SH, suggesting that it is indeed possible for people with PD and FSCD to maintain their SWB despite the chronic diseases they live with.

Investigating the predictors of SH is relevant to better understand the concept of happiness and to help people with a CND to maintain their happiness. It was unsurprising that depression predicted SH in both disease groups, as depression and anxiety are common in both. In PD, depression has an estimated prevalence of 40%^43^ and is multifactorial with both biological and reactive factors playing a role.^44^ The presence of depression is always associated with low SH^45^ and, therefore, an important factor to consider when supporting people with PD to maintain SWB. Depression is also prevalent in all forms of dystonia with approximately 31.5% of people with cervical dystonia, 29.2% with cranial dystonia, and 33.6% of people in a sample of people with mixed forms of dystonia^46^ reported depressive symptoms. These figures also demonstrate the importance of managing depression in dystonia in relation to SWB.

Resilience is the ability to bounce back from challenging circumstances.^47^ Resilience was a predictor of happiness in people with PD, suggesting that the greater the resilience to the impact of symptoms, the lower the impact of symptoms. The role of extraversion as a predictor of SH in people with PD is even more intriguing. People with high levels of extraversion tend to be happier by nature^48^ and as a result, extraversion has been repeatedly shown to predict happiness^49^, ^50^ as it promotes sociability. The results of this study suggest that extraversion is a personality trait that potentially supports people with PD to maintain their levels of SH as it may protect against the isolating aspects of the disease.

The hope scale was used as a measure of engagement with life as it measures an individual's ability to set goals (measured by agency) and finds a way to pursue them (measured by pathways). Resilience was the only predictor of hope pathways for people with FSCD, whereas in people with PD while disease duration, self‐esteem, and pain/discomfort predicted trait hope pathways, disease duration, self‐esteem, and anxiety predicted trait hope agency. Disease duration was a negative predictor of hope pathways and hope agency in PD. It is possible that the progressive nature of PD makes disease duration predict hope in people with PD, but not in FSCD, although disease duration was longer in FSCD. It makes sense that as one's symptoms increase and worsen as engaging with life through goal setting and finding pathways to achieve goals become more challenging. This premise may also explain why pain and discomfort were also predictors of hope pathways. Anxiety negatively predicted hope agency. This is consistent with previous research showing that, although both agency and pathway thinking are important aspects of hope in the context of anxiety, agency thinking may be crucial because agency thinking, but not pathway thinking, increases in strength with age.^51^ In our study, self‐esteem predicted both hope agency and pathway thinking. Self‐esteem is a complex construct encompassing self‐liking, self‐competency, and self‐confidence and has already been related to hope.^52^

However, there were no differences between the groups in the two hope scores. This lack of group difference in the hope scores may be because people with PD and FSCD are similar to other people with CND and are setting goals and finding pathways, but they are adjusting how many goals they set and how they find their pathways, which is something the scale is not sensitive enough to assess. This aspect of engagement is, therefore, worthy of further investigation as goal setting and engagement with life is a core aspect of well‐being and can help people living with CND to maintain daily engagement and standards of task performance and achievement.^53^

The lack of difference between the groups' search for meaning in life scores suggests that people continue to search for ways to make their lives meaningful irrespective of their state of health. What is interesting is that people with FSCD had lower presence of meaning in life than people with PD. This might be because of the fact that age at disease onset is lower in FCSD than PD. Namely, people generally tend to report greater meaning in life in later life,^14^ because they have more time to find their purpose or because they have more time to reflect on what is purposeful. This earlier disease onset may have affected their ability to find meaning in their lives more effectively. Anxiety was the only factor that predicted meaning in life search score in PD. This has already been shown in previous research in which high search for meaning and low presence of meaning was associated with high anxiety.^54^ This suggests that anxiety might act as a driving force in the search for meaning, which could later increase the presence of meaning in life.

Only self‐esteem and loneliness featured as a significant covariates for SWL in the between group analysis. We would have also expected social support to have featured, because loneliness and social support are interrelated, and social support can mediate the effects of loneliness.^55^ We also would have expected these covariates to have predicted SWL in FSCD. The link between life satisfaction and loneliness is well documented because most people feel the need to belong, and a sense of belonging brings life satisfaction.^56^ Loneliness is a subjective experience whereby the individual feels a deficit in the quantity or quality of the social relationships they experience.^57^ Living with PD or FSCD can impede such a sense of belonging for various reasons. Freezing and falling, including fear of falling, tremor, and medication‐induced dyskinesias, are some of the symptoms of PD^7^, ^58^ that can cause people with PD to withdraw socially because of low self‐esteem and gradually feel socially isolated.^59^ Similarly, the clinical effects of FSCD include involuntary abnormal postures and repetitive movements that can cause some people to have difficulties in social situations because of a sense of disfigurement, poor body image, and low self‐esteem.^9^, ^60^, ^61^ Self stigmatization is also high in focal dystonias such as cervical dystonia and spasmodic dysphonia,^62^, ^63^ which can lead to social avoidance and social isolation. Hence, high self‐esteem is a positive predictor of life satisfaction, and social isolation and loneliness predict poor life satisfaction.^64^

Self‐esteem predicted SWL in people with PD. Self‐esteem is a personality trait that is highly correlated with life satisfaction yet there is controversy over whether high self‐esteem comes as a consequence of good things happening,^65^ or if it is an innate motivator that helps the individual to seek out better life outcomes.^66^ Self‐esteem's role in SWL, hope pathways and agency suggests it plays an important role in living well with PD, because it has already shown how it can support people as they adapt to the changes in lifestyle imposed by disease.^67^

In considering the finding that anxiety negatively predicted SWL in PD, it is important to highlight the key role of anxiety in predicting an aspect of SWB in PD. Hope agency and search for meaning measure different aspects of engagement—one's agency with regards to goal achievement and the active search for meaning, which like SWL, were predicted by anxiety. These findings suggest that it is mental (ie, anxiety) rather than physical health that has the greatest impact on the SWB of people with PD.

Our finding of the lack of significant predictors of SWL in FSCD is consistent with past research that showed no difference in SWL between musicians with focal dystonia and HCs, because people with dystonia adapt to their disease and maintain life satisfaction.^68^ These findings suggest that people with FSCD have their own coping mechanisms, which help them to maintain their SWB.

There are several limitations of the study. We collected the data through a postal survey. Therefore, we were not able to objectively assess the motor status of the patients who participated. This did not allow for a subgroup analysis based on severity of motor symptoms or differences between early versus advanced stages of the disease particularly for PD. Second, most of the scales used in the study have not yet been validated in PD and FSCD. However, there are no specific scales to measure SWB in PD and FSCD, only leaving the possibility of using scales validated in the general population. It is evident that SWB in FSCD had fewer predictors than PD. This could be because of the biological and clinical differences between these two conditions, the longer disease duration of FSCD, but also differences in the sensitivity of the scales used to identify predictors of SWB in FSCD. For example, perceived disfigurement in dystonia, has a negative impact on body concept and self‐esteem leading to depression.^61^, ^69^, ^70^ Moreover, perceived stigma creates social embarrassment resulting in social avoidance and isolation.^71^, ^72^ Inclusion of measures such as perceived disfigurement and body concept might have resulted in better prediction of SWB in FSCD.

In summary, the results of our study show that PD and FSCD have lower life satisfaction and a lower meaning of life presence score than HC. We identified several significant predictors (reflecting mood, personality, disease duration, and pain/discomfort) of specific measures of SWB in PD. Depression was the only significant predictor of SH, and resilience was the only significant predictor of pathway to hope in FSCD. A better understanding of the determinants of SWB in PD and FSCD not only helps to better understand the way people respond to the disease, but also to adapt disease management strategies and provide psychological support to improve SWB in these patient groups. In the future, it would be interesting to include patients with other movement disorders such as essential tremor to allow further comparative analyses of the impact of CND on SWB.

## Author Roles

(1) Research project: A. Conception, B. Organization, C. Execution; (2) Statistical Analysis: A. Design, B. Execution, C. Review and Critique; (3) Manuscript: A. Writing of the First Draft, B. Review and Critique.

S.S.: 1A, 1B, 1C, 2C, 3A, 3B

D.G.: 2A, 2B, 2C, 3B

P.J.: 1C, 3B

K.B.: 1C, 3B

P.L.: 1C, 3B

M.J.: 1A, 1B, 1C, 2A, 2C, 3B

## Disclosures

**Ethical Compliance Statement**: Full ethical approval was granted by Health Research Authority and Health and Care Research Wales Ethics Committee (number 233401, REC Ref 18/LO/1368). As suggested by the Ethics Committee who reviewed and approved the proposal, the return of the completed booklet acted as implied consent to participate in the study. We confirm that we have read the Journal's position on issues involved in ethical publication and affirm that this work is consistent with those guidelines.

**Funding Sources and Conflicts of Interest:** D.G., was partially supported by the Slovenian Research and Innovation Agency (ARIS) project program P2‐0209 Artificial intelligence and intelligent systems. The authors declare that there are no conflicts of interest relevant to this work.

**Financial Disclosures for the Previous 12 Months:** The authors declare that there are no additional disclosures to report.

## Supporting information


**Data S1.** Below you will find a detailed description of the scales and questionnaires used in the study. Four subjective well‐being scales, one quality of life scale, two measures of mental health and six other measures, including personality measures, were used to assess and compare subjects with PD, focal/segmental cervical dystonia and healthy subjects.

## Data Availability

The data that support the findings of this study are available from the corresponding author upon reasonable request.
