# Supplementary material for: Subjective Well‐Being and Its Predictors in Parkinson's Disease and Dystonia: A Comparative Study
Source: Mov Disord Clin Pract. 2025 May 30;12(11):1792–800. doi: 10.1002/mdc3.70141 (PMC12625102; doi:10.1002/mdc3.70141)
Supplement: Supplementary file 1 — Data S1. Below you will find a detailed description of the scales and questionnaires used in the study. Four subjective well‐being scales, one quality of life scale, two measures of mental health and six other measures, including personality measures, were used to assess and compare subjects with PD, focal/segmental cervical dystonia and healthy subjects. [file MDC3-12-1792-s001.docx]

**Supplementary Material**

**Description of the scales and questionnaires used in the study**

***Measures of subjective wellbeing***

*1. Subjective Happiness Scale (SHS)*^1^ is a four-item scale with statements such as “in general I consider myself” and respondents are asked to circle a number between 1-7 where 1 signifies “not a very happy person” and 7 is “a very happy person.” Higher scores indicate higher levels of happiness.

*2. State Hope Scale (SHoS)*^2^ is a 12-item questionnaire on which respondents are asked to rate four items which assess the respondents approach to pathways (Trait Hope Pathways) to achieving goals such as “There are lots of ways around any problem,” and four questions which assess the individual’s agency (Trait Hope Agency) in achieving goals such as, “I energetically pursue my goals.” Four items are filler items. Higher scores indicate higher levels of search and agency.

*3. Meaning in Life Questionnaire (MLQ)*^3^ assesses two dimensions of meaning in life, search (Meaning in Life Search Score) and presence (Meaning in Life Presence Score) using a seven-point Likert scale. Respondents rate statements such as, “I am seeking a purpose or mission for my life,” which relates to the dimension of search and “My life has a clear sense of purpose,” which indicates presence of meaning in life. Higher scores indicate higher levels of meaning.

*4. Satisfaction with Life Scale (SLS)*^4^ is a five-item questionnaire which uses a seven-point Likert scale where respondents rate statements such as “in most ways my life is close to my ideal”. Higher scores indicate higher levels of life satisfaction.

***Generic quality of life measure:*** EQ-5D^5^ was completed by all participants to measure quality of life across five dimensions: mobility, self-care, usual activities, pain and discomfort and anxiety and depression using five levels of severity which are coded on a five-point scale where 1= no problem and 5= unable. The EQ-5D also contains the EQ Visual Analogue Scale (VAS) which is a 0-100 scale whereby 100 is the best health imaginable and 0 is the worst.

***Mental Health Measures***

*1. Hospital Anxiety and Depression scale (HADS)^6^* is a 14-item scale that contains two subscales, one for anxiety one for depression with a possible overall score of 21 for each sub-scale. For anxiety or depression subscales, scores of 0-7 have been deemed to be within the normal range, 8-10 is borderline and scores of 11 and above suggest ‘caseness’.

*2. UCLA Loneliness Scale (LS)^7^* is a 20-item scale of which nine are reverse scored measure levels of subjective loneliness. Items include “How often do you feel alone?” Respondents are asked to respond on a four-point scale where 1= Never and 4 = Always. Higher scores indicate higher levels of loneliness.

***Other Measures***

*1. Self-Esteem Questionnaire (SEQ)^8^* is a 10-item questionnaire of which 5 items are positively worded, such as, “I take a positive attitude towards myself.” Five items are negatively worded and reverse scored such as, “At times I think I am no good at all.” All items are scored on a four-point likert scale whereby 0=strongly disagree and 3=strongly agree. Scores ranging from 0-15 suggest low levels of self-esteem, 15-25 suggest normal levels of self-esteem and 25-30 suggest high levels of self-esteem.

*2. The Stigma Scale (SS)^9^* is a six-item scale assessing the extent to which illness has affected how they perceive themselves in relation to others and the extent to which they feel stigmatized by others. Answers are rated using a 4 point scale where 0=not at all and 3=definitely.

3. *Eysenck Personality Questionnaire (EPQ) short form^10^* contains 48 items, 12 for each of the traits of neuroticism, extraversion, and psychoticism, and 12 for the lie scale. Each question has a binary response, 'yes' or 'no'. For example, “Does your mood go up and down?” or “Are you a worrier?” The EPQ-SF measures four personality traits whereby higher scores indicate higher levels of trait psychoticism, extraversion, neuroticism and social desirability (Lie).

*4. Life Orientation Test (LOT)^11^* is a 10-item scale that measures levels of optimism. Three questions are positively worded such as, “In uncertain times I usually expect the best”, three items are negatively worded and reverse scored, such as, “If something can go wrong for me it will,” and there are four filler questions. Scores ranging from 0-13 suggest low levels of optimism, 14-18 suggest moderate levels of optimism and scores ranging from 19-24 suggest high levels of optimism.

*5. The Short Social Support Questionnaire (SSSQ)^12^*  measures the quantity (number of people) and quality (satisfaction with) of practical and emotional social support. Respondents are asked to name friends or relatives who support them practically or emotionally and are then asked to rate their levels of satisfaction for each dimension from 1- 6 where 1=very dissatisfied and 6=very satisfied. Higher scores indicate higher levels of perceived social support.

*6. Brief Resilience Scale (BRS)^13^* is a six-item scale in which 3 items are positively worded, and three items are negatively worded and reverse scored. Respondents are asked to reflect on statements such as “I tend to bounce back quickly after hard times” and then indicate to what extent the statement represents themselves using a five-point likert scale where 1=strongly disagree and 5=strongly agree. The higher the score the more resilient the respondent.

1. Lyubomirsky S, Lepper HS. A measure of subjective happiness: Preliminary reliability and construct validation. Social indicators research 1999;46(2):137-155.

2. Snyder CR, Sympson SC, Ybasco FC, Borders TF, Babyak MA, Higgins RL. Development and validation of the State Hope Scale. Journal of personality and social psychology 1996;70(2):321.

3. Steger MF, Frazier P, Oishi S, Kaler M. The meaning in life questionnaire: Assessing the presence of and search for meaning in life. Journal of counseling psychology 2006;53(1):80.

4. Diener E, Emmons RA, Larsen RJ, Griffin S. The Satisfaction With Life Scale. J Pers Assess 1985;49(1):71-75.

5. Herdman M, Gudex C, Lloyd A, et al. Development and preliminary testing of the new five-level version of EQ-5D (EQ-5D-5L). Quality of life research 2011;20(10):1727-1736.

6. Zigmond AS, Snaith RP. The hospital anxiety and depression scale. Acta Psychiatr Scand 1983;67(6):361-370.

7. Russell D, Peplau LA, Cutrona CE. The revised UCLA Loneliness Scale: concurrent and discriminant validity evidence. Journal of personality and social psychology 1980;39(3):472.

8. Rosenberg M. Rosenberg self-esteem scale (SES). Society and the adolescent self-image 1965.

9. MacDonald L, Anderson H. Stigma in patients with rectal cancer: a community study. Journal of Epidemiology & Community Health 1984;38(4):284-290.

10. Eysenck SB, Eysenck HJ, Barrett P. A revised version of the psychoticism scale. Personality and individual differences 1985;6(1):21-29.

11. Scheier MF, Carver CS. Optimism, coping, and health: assessment and implications of generalized outcome expectancies. Health psychology 1985;4(3):219.

12. Jahanshahi M, Marsden CD. Personality in torticollis: a controlled study. Psychological medicine 1988;18(2):375-387.

13. Smith BW, Dalen J, Wiggins K, Tooley E, Christopher P, Bernard J. The brief resilience scale: assessing the ability to bounce back. International journal of behavioral medicine 2008;15(3):194-200.
